# Suicidal ideation and psychological dating violence victimization—A short report

**DOI:** 10.3389/fpsyt.2023.1105654

**Published:** 2023-06-02

**Authors:** Jackson W. S. Gasperecz, Elizabeth Baumler, Leila Wood, Jeff R. Temple

**Affiliations:** Center for Violence Prevention, University of Texas Medical Branch at Galveston, Galveston, TX, United States

**Keywords:** suicidal ideation, intimate partner violence, dating violence, psychological abuse, suicidality

## Abstract

As the second leading cause of death among Americans aged 10 to 34, suicide is a serious public health concern. One potential predictor of suicidality is dating violence (DV) victimization, such as any physical, psychological, or sexual abuse by a current or former intimate partner. However, little longitudinal data exists on the relationship between suicidal ideation and DV. To address this gap in knowledge, we leverage data from two years of our longitudinal study *Dating It Safe*. Specifically, we examine whether physical and psychological DV victimization is associated with subsequent suicidal ideation in our ethnically diverse sample of young adults (*n* = 678; mean age = 25 at Wave 9; 63.6% female). While physical DV victimization was not linked to suicidal ideation over time, psychological DV victimization was (*χ*^2^ = 7.28, *p* = 0.007 for females; *χ*^2^ = 4.87, *p =* 0.027 for males). That psychological abuse was potentially as or more impactful than physical violence is consistent with the broader literature on the deleterious impacts of psychological violence, as well as the limited longitudinal literature looking at DV and suicidality specifically. These findings reinforce the notion that psychological abuse is as consequential as physical violence in the long-term, has unique impacts on mental health, and points to the need for both suicide and violence intervention programs to address this form of dating violence victimization.

## Introduction

Over the past two decades, the US has witnessed a steady increase in the rate of suicides (1). In 2017, the suicide rate for ages 15 to 19 (17.9 per 100,000 males and 5.4 in 100,000 females) and 20 to 24 (17 per 100,000 in males and females combined) increased to its highest point since 2000 (2). Emergent data suggests these rates may have increased during the COVID-19 pandemic (3). Suicidal ideation, or suicidal thoughts, describes a range of ideas, ruminations, or preoccupations with ending one’s own life (4). To date, there is little known about the transition between thinking about suicide and attempting suicide (5). Meta-analytic data have demonstrated that while depression and hopelessness are strong correlates of suicidal ideation, these variables are weakly associated with suicide attempts among those who experience suicidal ideation (6). Different theories have been proposed to explain suicide capability, but research has yet to determine a single strong predictor of suicide attempts among those who experience suicidal ideation (5).

Dating violence (DV) can be described as a pattern of controlling, harmful, and coercive behavior within an intimate relationship (7) and can encompass physical abuse, sexual violence, and psychological aggression (8). While the former two have been linked to several health consequences (e.g., headaches, back pain, digestive problems) (9), the more prevalent psychological DV (9, 10) has also been consistently linked to negative outcomes, including Post-Traumatic Stress Disorder (PTSD) and depression (11–15). Suicides may account for close to 30% of DV related deaths (16). In a systematic review of existing literature, depressive symptoms were associated with DV and DV was associated with suicide attempts (17). Further, while both physical and psychological DV victimization have been linked to suicidal ideation (18–21), the latter is more robustly linked (22–24). However, the bulk of this research is cross-sectional in nature [e.g., (22)], thus limiting our ability to detect any longitudinal relationships between these variables.

To address this gap in knowledge, we examine the longitudinal link between DV victimization and suicidal ideation among a large ethnically diverse sample of young adults. Consistent with the mostly cross-sectional literature, we hypothesize that physical and psychological DV victimization will be significantly associated with subsequent suicidal ideation; however, psychological DV victimization will be a more salient predictor of suicidality.

## Methods

### Participants

We used data from an ongoing longitudinal study of the risk and protective factors of dating violence (i.e., *Dating It Safe*). At baseline (spring 2010), participants were recruited from seven public high schools in five Houston-area school districts (*n* = 1,042; 56% female; mean age = 15.1). For the current analyses, we focus on data from Waves 9 (collected in 2020, when participants ranged in age from 22 and 25) and 10 (collected in 2021; age range = 23 to 26) when the relevant variables were assessed. We included participants who completed both assessment waves (*n* = 678, 63.6% female). Detailed study methodology can be found in numerous prior publications [e.g., (25–27)].

### Measures

We assessed suicidal ideation with the following yes/no question asked at both Waves 9 and 10: “Have you thought about suicide in the PAST YEAR?.” “Yes” responses were coded with a “1” and “No” responses were coded with a “0.” DV victimization was measured with the Conflict in Adolescent Dating Relationship Inventory [CADRI; (28)], and included 4 items assessing physical DV victimization (e.g., “He/She kicked, hit, or punched me”) and 10 items assessing psychological DV victimization (10 items; e.g., “He/She deliberately tried to frighten me”). Respondents who indicated “yes” to any physical DV item were considered victims. Given the high endorsement of psychological DV and, as we and others have done in prior studies [e.g., (29, 30)], respondents had to endorse 4 or more items to be considered victims of psychological DV. The Cronbach’s *α* for physical and psychological DV victimization in the current study was 0.86 and 0.87, respectively.

### Data analysis

Descriptive analyses were used to provide prevalence estimates both in total and by gender. Associations between psychological, physical DV victimization and suicidal ideation were assessed using a chi-square statistic. Type I error rates of 0.05 were used to identify statistical significance. While we were primarily interested in prospectively examining DV victimization on suicidality, we also investigated the reverse association (i.e., suicidality to DV).

## Results

As shown in Table 1, psychological DV victimization at Wave 9 was associated with suicidal ideation at Wave 10 for both females and males (*χ*^2^ = 7.28, *p* = 0.007; *χ*^2^ = 4.87, *p =* 0.027, respectively). Significant associations did not emerge between physical DV victimization and suicidal ideation. In addition, we did not find a prospective association between suicidal ideation at Wave 9 and subsequent physical or psychological DV at Wave 10.

**Table 1:** Prevalence of dating violence victimization and suicidal ideation among participants.

Measure	*N*	%
Physical DV Victimization at Wave 9	104	15.5%
Male	43	17.7%
Female	61	14.3%
Psychological DV Victimization at Wave 9	268	40.5%
Male	90	37.7%
Female	178	41.3%
Suicidal Ideation at Wave 10	142	21.2%
Male	46	18.9%
Female	96	22.4%

Respondents who indicated “Yes” to one or more physical DV items were considered victims. Respondents who endorsed 4 or more psychological DV items were considered victims of psychological DV.

## Discussion

While psychological DV has been historically minimized with respect to health impacts and legal implications [e.g., (31, 32)], researchers continually find it to be both prevalent and consequential—perhaps even more damaging than physical violence [e.g., (33, 34)]. In our longitudinal study of the association between DV victimization and suicidal ideation, we found that psychological DV, and not physical DV, was associated with subsequent suicidality. Findings support the mostly cross-sectional literature that psychological abuse may be especially important in considering long-term mental health consequences of DV (19, 22, 23). This result may not be surprising when considering the characteristics of psychological harm and its overlap with coercive control. The latter is characterized by dominance, surveillance, and manipulation and it is intended to elicit fear and alienate victims from their social support networks [e.g., (35)]. Indeed, research has shown that coercive control drives the relationship between psychological abuse and suicidal ideation (20, 23, 36).

Our finding that psychological DV is a potentially important predictor of suicidal ideation is also consistent with the broader literature. In a sample of women residing in domestic violence shelters, Dokkedahl et al. (37) found that psychological abuse, relative to physical and sexual violence, was a stronger correlate of accompanying mental health problems, including PTSD and complex PTSD. Another study found that psychological abuse was a predictor of severe physical injuries among a sample of help-seeking battered women (38). Our study joins other scholarly work asserting the need for robust mental and social supports for DV victims to prevent suicidal ideation.

To be clear, our findings should not be interpreted that physical violence is not important or injurious. Indeed, other longitudinal studies have found that physical DV victimization can be a serious risk factor for suicidality among adolescents and young adults (22, 39–42) and is a primary indicator for DV related homicide. Instead, researchers, practitioners, and the legal system need to consider psychological violence as a unique threat to the health and safety of DV victimizations, especially as it relates to suicide and suicidal ideation. Mental health practitioners focused on DV may benefit from additional training in assessing and addressing suicidal ideation. Further, assessments for suicidality in mental health settings should incorporate DV, and particularly psychological violence, as a risk factor for suicidal ideation. As Decker et al. (43) propose, an intersectional and socioecological framework using crosscutting interventions is needed to address the multi-directional relationship between interpersonal violence (such as DV) and self-harm risks.

From a theoretical perspective, the interpersonal-psychological theory of suicide (IPTS) offers a potential explanation for the link between psychological DV victimization and suicidal ideation (44, 45). The IPTS posits that suicidal ideation occurs as the result of perceived burdensomeness and thwarted belongingness, both of which may be a consequence of psychological DV. Wolford-Clevenger et al. (45) suggest that psychological aggression may drive these characteristics of suicidal ideation by isolating victims and increasing their emotional and financial dependence on their abusive partners (46, 47). Psychological violence (e.g., repeated insults, humiliation, and intimidation) likely contribute to sustained feelings of worthlessness and burden. Further, the perception of burden and potential harm from disclosing DV to friends and family who might offer emotional support may preclude victims from reaching out for help to reduce isolation. Several limitations of the present study should be considered. First, we relied on retrospective self-reports of suicidal ideation and DV victimization, which may have impacted the accuracy of our results (48). Second, we limited our study to DV victimization. Given the potential link between DV perpetration and suicidal ideation (23, 49), future longitudinal research should examine this relationship. Finally, our analysis does not consider economic, community, or social factors that may accelerate or mitigate risk for suicidal ideation. Despite these limitations, our findings add to the growing literature that psychological abuse is uniquely consequential to suicidality among young adult DV victims, regardless of gender.

## Data availability statement

Publicly available datasets were analyzed in this study. This data can be found here: https://www.icpsr.umich.edu/web/pages.

## Ethics statement

The studies involving human participants were reviewed and approved by UTMB Institutional Review Board at UTMB. The patients/participants provided their written informed consent to participate in this study.

## Author contributions

JG wrote an initial draft of the report, amended, and finalized the report. JT provided two rounds of edits and suggestions with regards to the introduction, methods, and discussion. EB led data analysis and composed the results section. LW provided a final round of edits of the introduction and discussion. All authors contributed to the article and approved the submitted version.

## Conflict of interest

The authors declare that the research was conducted in the absence of any commercial or financial relationships that could be construed as a potential conflict of interest.

## Publisher’s note

All claims expressed in this article are solely those of the authors and do not necessarily represent those of their affiliated organizations, or those of the publisher, the editors and the reviewers. Any product that may be evaluated in this article, or claim that may be made by its manufacturer, is not guaranteed or endorsed by the publisher.
